# Bacterial artificial chromosomes improve recombinant protein production in mammalian cells

**DOI:** 10.1186/1472-6750-9-3

**Published:** 2009-01-14

**Authors:** Leander Blaas, Monica Musteanu, Robert Eferl, Anton Bauer, Emilio Casanova

**Affiliations:** 1 Ludwig Boltzmann Institute for Cancer Research (LBI-CR), Währinger Str. 13a, A-1090 Vienna, Austria; 2 f-star, Gastgebgasse 5-13, A-1230 Vienna, Austria

## Abstract

**Background:**

The development of appropriate expression vectors for large scale protein production constitutes a critical step in recombinant protein production. The use of conventional expression vectors to obtain cell lines is a cumbersome procedure. Often, stable cell lines produce low protein yields and production is not stable over the time. These problems are due to silencing of randomly integrated expression vectors by the surrounding chromatin. To overcome these chromatin effects, we have employed a Bacterial Artificial Chromosome (BAC) as expression vector to obtain stable cell lines suitable for protein production.

**Results:**

In this work, we explore the efficacy of a Bacterial Artificial Chromosome based vector applied to production of the constant region of the human IgG1. Direct comparison of bulk HEK 293 cell cultures generated with a "conventional" vector or with a BAC-based vector showed that the BAC-based vector improved the protein yield by a factor of 10. Further analysis of stable cell clones harboring the BAC-based vector showed that the protein production was directly proportional to the number of integrated BAC copies and that the protein production was stable for at least 30 passages.

**Conclusion:**

Generation of stable cell clones for protein production using Bacterial Artificial Chromosomes offers a clear advantage over the use of conventional vectors. First, protein production is increased by a factor of 10; second, protein production is stable overtime and third, generation of BAC-based expression vectors does not imply a significant amount of work compare to a conventional vector. Therefore, BAC-based vectors may become an attractive tool for protein production.

## Background

Recombinant protein production in mammalian cells is an important topic in biotechnology [1]. One of the critical steps in the production of recombinant proteins is the isolation of stable single cell clones expressing high levels of the protein of interest. Commonly, this is achieved by random genomic integration of a vector containing a promoter, a gene of interest and a selectable marker. Although this method is simple and straight forward, it lacks of reproducibility. Expression from such vectors is substantially influenced by the surrounding chromatin to the integration site and tends to be silenced over time. This makes the selection of suitable clones a tedious and time consuming procedure [1]. Several strategies have been developed to overcome the positional effects of the adjacent chromatin. For example, "anti-repressor" elements flanking the vectors [2] have been used or vectors have been integrated specifically into chromosomal loci with open chromatin [3]. Ideally, a vector for recombinant protein production should display three features: 1) expression should be independent of the integration site in the genome, 2) expression should correlate with the number of integrated transgene copies and 3) expression should be maintained over time. Interestingly, large vectors that fulfill these criteria such as Bacterial Artificial Chromosomes (BACs) have been widely used for generation of transgenic mice [4] but not for production of recombinant proteins. BACs are vectors derived from the F factor of *E. coli *that are maintained as low copy replicons. BACs offer a clear advantage compared to "classical expression vectors": Due to their large cloning capacity (up to 300 Kb), BACs can accommodate most (if not all) of the elements that are responsible for the expression of a gene of interest. Thus, BACs can be considered as complete expression units. Consequently, expression from BACs based vector is less affected by the surrounding chromatin to their insertion site in a host genome. In this sense, BACs containing genes that are considered as open chromatin (highly transcribed), such as *Rosa26*, *β-actin*, *Gapdh *etc. are attractive tools in the field of eukaryotic recombinant protein production. On the other hand, due to their large size, BACs can not be manipulated using traditional cloning techniques. Modification of BACs is done via homologous recombination in *E. coli *(recombineering), however, there are several existing methods that allow to modify a BAC via homologous recombination in *E. coli*, thus making the use of BACs as expression vectors a relative simple task [5-7]. In this work, we explore the suitability of a BAC containing the *Rosa26 *locus as expression vector applied to the production of the Fc fragment of the constant region of human IgG1 in HEK 293 cells.

## Methods

### Plasmids and cell culture

The CAGGS Fc expression vector was assembled by conventional cloning methods and is flanked by two attB sites (ɸC31 integrase recognition sites). The *Rosa26*^BAC CAGGS Fc ^BAC vector was generated as previously described [8]. Briefly, the CAGGS Fc vector was recombined into a BAC containing the *Rosa26 *locus using ɸC31 mediated cassette exchange into the exon 2 of the *Rosa26 *antisense transcript.

To establish the bulk cultures, 24 μg CAGGS Fc and *Rosa26*^BAC CAGGS Fc ^BAC vectors were linearized with NotI and transfected into HEK 293 cells using Lipofectamine 2000 (Invitrogen). Two days after transfection, G418 (800 μg/ml) was added to the media (DMEM high glucose, 10% FCS, supplemented with glutamine, pyruvate and non essential aminoacids). Selection was carried out over 14 days. Thereafter, all the cultures were grown in the absence of G418 and Fc protein production in the bulk cultures was measured 1 week later.

### Human IgG1-Fc protein determination

5 × 10^5 ^cells were seeded into each single well of a 6 well plate in 2 ml of medium. 72 hours after seeding, the Fc protein concentration was measured in the supernatants using an ELISA assay. For the ELISA, goat anti-human-IgG (Fc specific) F(ab')2 fragment (Sigma I-3391) was adsorbed at 1 μg/ml onto microwells of a Maxisorp plate over night at 4°C, followed by blocking with 5% BSA in PBS for 1 h at 25°C. After washing, the samples and the standard, respectively, were added in dilution series to the blocked microwells and incubated for 1 h at 25°C. The plate was washed and bound Fc was detected by Protein A – HRP (SIGMA P-8651) diluted 1:70000 in PBS/BSA followed by staining with TMB substrate solution (Sigma T0446). The ELISA was measured at 450 nm with the reference wavelength 630 nm.

### *Rosa26*^BAC CAGGS Fc ^copy number analysis

The *Rosa26*^BAC CAGGS Fc ^culture was enriched twice using eYFP FACS sorting. Single clones were established using a dilution technique. Cells were seeded in 96 well plates at 0.5 cells per well. Clones were expanded progressively up to 10 cm dish, at this point (confluent 10 cm dish), cells were considered passage 1. During subcloning, expansion and subsequence analysis of the cultures no selective pressure was used. The number of transgene copies in the single cell clones (at passage 1) was quantified by real time PCR with genomic DNA as template. The *Rosa26 *BAC was amplified with oligos RosaF 5' TCTTGTCCTTTTACCTCCCTTGTA RosaR 5' GAACATATTCAAAACACCAGGATTT. These oligos recognize a sequence that is identical in the *Rosa26 *BAC (from murine origin) and in the endogenous *Rosa26 *locus (HEK cells, from human origin). The *β-actin *locus was amplified with oligos, actinF 5' TCATGTTTGAGACCTTCAACACC and actinR 5' GATCTTCATGAGGTAGTCAGTCAGGT as internal control to normalize the amount of genomic DNA used.

## Results

**Figure 1:**
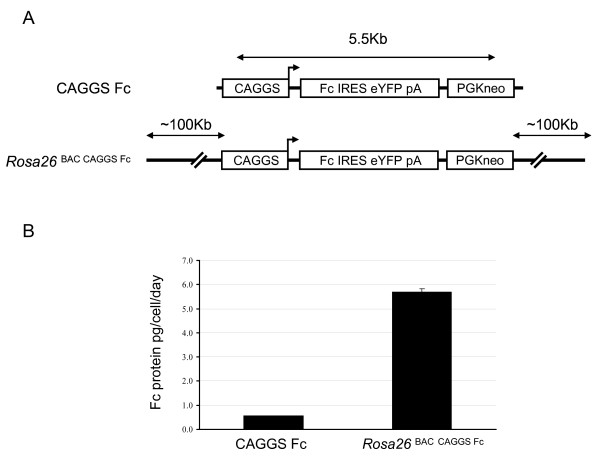
**Use of a Bacterial Artificial Chromosome (BAC) as vector backbone for recombinant protein production**. **A) **Schematic representation of the constructs used for protein production. CAGGS Fc is a conventional expression vector containing a CAGGS promoter, the Fc region of the human IgG1 gene, an IRES-eYFP-SV40 polyA reporter element and a PGK neomycin cassette. The *Rosa26*^BAC CAGGS Fc ^construct was generated by recombining the CAGGS Fc vector into a BAC containing the *Rosa26 *locus. The CAGGS Fc vector was placed into the exon 2 of antisense transcript of the *Rosa26 *locus with 100 kb upstream and downstream sequence. **B) **Comparison of the efficacy in protein production between a conventional vector and a BAC-based vector. The Fc yield was analyzed in HEK 293 bulk cultures generated with the CAGGS Fc and *Rosa26*^BAC CAGGS Fc ^vectors. The CAGGS Fc vector gave a yield of 0.5 pg/cell/day, while the BAC-based vector gave a yield of 5.7 pg/cell/day. Measurements were performed in triplicate. Error bars represent the standard deviation.

In order to test the efficacy of BACs in the production of recombinant proteins, we have generated two expression vectors: The CAGGS Fc vector, which consists of a CAGGS promoter [9], the Fc fragment (i.e. hinge, C_H_2 and C_H_3) of human IgG1 as gene of interest (Fc) containing a leader peptide for secretion [10], an IRES/eYFP reporter and a PGK-neomycin cassette. The second vector, *Rosa26*^BAC CAGGS Fc^, consists of the CAGGS Fc vector that has been recombined into a BAC backbone containing the *Rosa26 *locus (Figure 1A). HEK 293 cells were transfected with the CAGGS Fc and the *Rosa26*^BAC CAGGS Fc ^vectors. After 14 days of G418 selection we obtained more than 2000 G418 resistant clones for the CAGGS Fc vector and 200–300 with the *Rosa26*^BAC CAGGS Fc ^vector. Cells from each culture were pooled and two bulk cultures for the CAGGS Fc and *Rosa26*^BAC CAGGS Fc ^vectors respectively were established. Analysis of the Fc protein production in the supernatants showed a yield of 0.5 and 5.7 pg/cell/day in the CAGGS Fc and the *Rosa26*^BAC CAGGS Fc ^bulk cultures, respectively (Figure 1B), demonstrating that the use of a BAC-based vector improves the protein production substantially.

**Figure 2:**
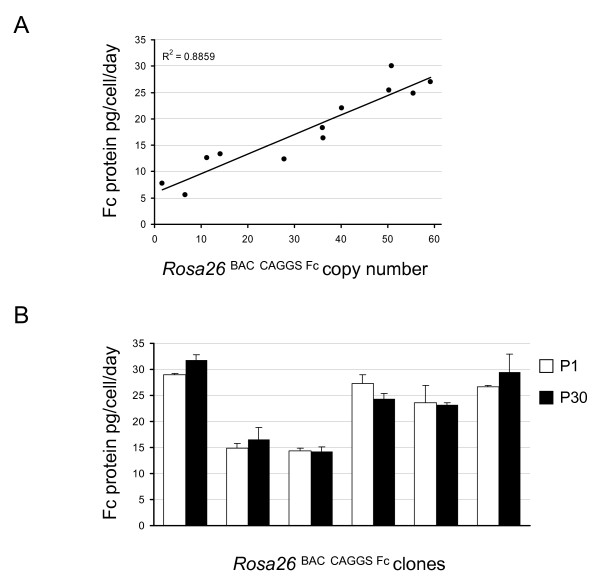
**Protein expression using a BAC as vector backbone is transgene copy-number dependent and it is stable over time**. **A) **Correlation between protein production and integrated BAC transgene copies. Analysis of 12 single clones showed a copy number range from 1 to 55 for the *Rosa26*^BAC CAGGS Fc ^vector with a Fc yield of 5.5 to 30 pg/cell/day. The protein production was directly proportional to the BAC transgene copy number. **B) **Protein production is stably maintained during culture passaging. The yield of Fc protein was measured at passage 1 and passage 30 in 6 subclones isolated from the *Rosa26*^BAC CAGGS Fc ^cultures. No obvious differences were found. Error bars represent the standard deviation.

Next, we analyzed the correlation between the number of transgene copies and the Fc protein yield. 12 subclones were established from the of *Rosa26*^BAC CAGGS Fc ^culture that harbored 1 to 55 copies of the transgene. Protein yield in the supernatants of these cultures correlated with the transgene copy numbers and ranged from 5.5 to 30 pg/cell/day (Figure 2A). The correlation coefficient R^2 ^between the copy number of the BAC vector and protein production was 0.88. This suggests that the protein production is proportional to the number of integrated transgene copies when using a BAC-based expression vector.

Finally, we investigated long-term protein production over time and increasing passage numbers. 6 subclones from the *Rosa26*^BAC CAGGS Fc ^culture were grown for 30 passages and protein production was analyzed. The yield of the Fc protein was not significantly decreasing from passage 1 to passage 30 (Figure 2B) indicating that BAC-based vectors provide stable long-term production of recombinant proteins.

## Discussion

In this work, we have used a BAC containing the *Rosa26 *locus for protein production. The *Rosa26 *locus is considered to be a region of open chromatin (transcriptionally active) and has been successfully used to express genes of interest in transgenic mice [11]. With this approach, we have shown that a BAC-based vector improves recombinant protein production substantially when compared to a conventional vector. Further improvements of BAC-based vectors for recombinant protein production could include the use of endogenous/natural promoters that are highly active in the producer cell. For example, a transcriptional profiling of HEK 293 cells has identified strong expression levels of the *Rpl23a *gene which encodes a ribosomal protein [12]. Therefore, the use of a BAC containing the *Rpl23a *locus in HEK 293 cells could further improve recombinant protein production.

## Conclusion

The *Rosa26 *BAC fulfills the ideal characteristic of an expression vector applied to protein production in stable cell lines. It increases protein production by a factor of 10 when compared to a conventional vector and it confers copy number dependent and stable protein expression. These results suggest that BAC-based expression vectors overcome negative effects of the surrounding chromatin at the transgene integration site. Consequently, BAC-based expression vectors represent an important tool to improve recombinant protein production.

## Authors' contributions

LB and M M performed experiments. AB and RE performed and designed experiments. EC designed experiments and wrote the manuscript.
